# Increased coronary arteriolar contraction to serotonin in juvenile pigs with metabolic syndrome

**DOI:** 10.1007/s11010-019-03589-6

**Published:** 2019-07-27

**Authors:** Isabella Lawandy, Yuhong Liu, Guangbin Shi, Zhiqi Zhang, Laura A. Scrimgeour, Vasile Pavlov, Reed Jaworski, Frank W. Sellke, Jun Feng

**Affiliations:** 1 Division of Cardiothoracic Surgery, Cardiovascular Research Center, Rhode Island Hospital, Alpert Medical School of Brown University, Providence, RI USA; 2 Division of Cardiothoracic Surgery, Cardiovascular Research Center, Rhode Island Hospital, Alpert Medical School of Brown University, Coro West, Room 5.229, 1 Hoppin Street, Providence, RI 02903 USA

**Keywords:** Serotonin, Microvascular constriction, Coronary arterioles, Metabolic syndrome, Juvenile obesity, Pigs

## Abstract

Metabolic syndrome (MetS) is associated with alterations in coronary vascular smooth muscle and endothelial function. The current study examined the contractile response of the isolated coronary arterioles to serotonin in pigs with and without MetS and investigated the signaling pathways responsible for serotonin-induced vasomotor tone. The MetS pigs (8-weeks old) were fed with a hyper-caloric, fat/cholesterol diet and the control animals (lean) were fed with a regular diet for 12 weeks (*n* = 6/group). The coronary arterioles (90–180 μm in diameter) were dissected from the harvested pig myocardial tissues and the in vitro coronary arteriolar response to serotonin was measured in the presence of pharmacological inhibitors. The protein expressions of phospholipase A2 (PLA_2_), TXA_2_ synthase, and the thromboxane-prostanoid (TP) receptor in the pigs’ left ventricular tissue samples were measured using Western blotting. Serotonin (10^−9^–10^−5^ M) induced dose-dependent contractions of coronary-resistant arterioles in both non-MetS control (lean) and MetS pigs. This effect was more pronounced in the MetS vessels compared with those of non-MetS controls (lean, *P* < 0.05]. Serotonin-induced contraction of the MetS vessels was significantly inhibited in the presence of the selective PLA_2_ inhibitor quinacrine (10^−6^ M), the COX inhibitor indomethacin (10^−5^ M), and the TP receptor antagonist SQ29548 (10^−6^ M), respectively (*P* < 0.05). MetS exhibited significant increases in tissue levels of TXA_2_ synthase and TP receptors (*P* < 0.05 vs. lean), respectively. MetS is associated with increased contractile response of porcine coronary arterioles to serotonin, which is in part via upregulation/activation of PLA_2_, COX, and subsequent TXA_2_, suggesting that alteration of vasomotor function may occur at an early stage of MetS and juvenile obesity.

## Introduction

Approximately 50 million Americans are affected by metabolic syndrome (MetS), which is described by a cluster of metabolic abnormalities including hypercholesterolemia, hypertriglyceridemia, and hypertension. These patients experience a significantly higher risk for coronary artery disease and type 2 diabetes [1–4]. MetS is associated with alterations in endothelial function, vasomotor control, and the dysregulation of coronary blood flow, which in turn could underlie increased cardiovascular morbidity and mortality in these patients [5–11]. Notably, these changes occur prior to overt atherosclerotic disease, and have been associated with left ventricular dysfunction in human and animal models of MetS [9–12].

Vascular diseases are the principal contributors to the increased morbidity and mortality associated with MetS [13, 14]. Endothelial dysfunction is considered to be a major risk factor of cardiovascular complications in patients with MetS and specifically contributes to the exacerbation of vasospasm, myocardial dysfunction, and low cardiac output syndrome. Given that MetS affects as much as 27% of the population of the United States and is increasing dramatically in prevalence, this remains a considerable clinical problem.

The mechanism behind the alteration in the MetS induced microvascular dysfunction is incompletely understood, but may involve the dysregulation of chemical and electrical signaling in the coronary microcirculation. The endothelium controls the tone of the underlying vascular smooth muscle through the release of various vasoactive substances, including nitric oxide (NO), prostacyclin (PGI_2_), thromboxane A_2_ (TXA_2_), serotonin (5-hydroxytryptamine, 5-HT), and endothelium-dependent hyperpolarizing factors (EDHF) [9, 10, 15, 16].

Serotonin is a vasoactive amine responsible for alterations in vessel contractile response in numerous organs. Previous studies by Metais and colleagues have shown that serotonin is responsible for mediating the increased coronary contraction associated with myocardial dysfunction after cardioplegic ischemia and cardiopulmonary bypass (CP/CPB), as well as with the change from serotonin-induced coronary vasodilation before CP/CPB [15, 16]. A possible cause for this may be the activation of phospholipase A_2_ (PLA_2_) by serotonin [16].

It has previously been shown that serotonin activates PLA_2_, which leads to the release of inflammatory mediators in the human heart [15–17]. This occurs via serotonin’s release of arachidonic acid, which then reacts with cyclooxygenase (COX) to form the vasoconstrictive agent TXA_2_, which likely contributes to the subsequent vasoconstriction seen following CP/CPB and cardiac surgery [15–17]. Therefore, we hypothesized that early stage of MetS may cause significant increase in the coronary arteriolar response to serotonin and the overexpression/activation of PLA_2_ and TXA_2_ may contribute to altered coronary microvascular reactivity. Thus, this study examined the contractile response of the isolated coronary arterioles to serotonin in pigs with or without MetS and investigated the signaling pathways responsible for serotonin-induced vasomotor tone.

## Methods

### Pig model of metabolic syndrome

Twelve Yorkshire swine (8-week old) arrived at our facility and after a week of acclimation, were separated into two groups: the normal (lean) diet group (*n* = 6) and the high-fat diet group (*n* = 6). The non-MetS control group (lean) received 500 g/day of regular chow for 12 weeks. The high-cholesterol animals received 500 g/day of high-cholesterol chow consisting of 4% cholesterol, 17.2% coconut oil, 2.3% corn oil, 1.5% sodium cholate, and 75% regular chow (Sinclair Research, Columbia, MO) for 12 weeks. The high-fat diet used in our animal model administered for 12 weeks has been shown to induce obesity, hyperlipidemia, high blood pressure, insulin resistance, and glucose intolerance, all of which are components of MetS [18–21]. After 12 weeks, the pigs were then euthanized by exsanguination following removal of the heart while under deep isoflurane anesthesia. The harvested myocardial tissues and segments of the coronary arteries in the LAD were placed in cold (4 °C) Kreb solution for vascular physiological study.

All experiments were approved by the Rhode Island Hospital Institutional Animal Care and Use Committee. Animals were cared for in compliance with the “Principles of Laboratory Animal Care” formulated by the National Society for Medical Research and the “Guide for the Care and Use of Laboratory Animals” (NIH publication number 5377-3, 1996).

### Microvessel reactivity

Coronary arterioles (90- to 180-μm internal diameters) of the left anterior descending (LAD) territory were dissected from the harvested left ventricular (LV) tissue samples. Microvessel studies were performed by in vitro organ bath video-microscopy as described previously [15, 16]. After a 60-min stabilization period, the microvessels were constricted with serotonin (10^−9^–10^−5^ M) in the absence or presence of the selective PLA_2_ inhibitor quinacrine (10^−6^ M) or the COX inhibitor indomethacin (10^−5^ M) or the selective thromboxane-prostanoid (TP) receptor antagonist SQ29548 (10^−6^ M). Some of the microvessels were constricted with TXA-2 analog U46619 (10^−9^–10^−6^ M). One or three interventions were performed on each vessel. The order of drug administration was random.

### Immunoblot

The methods for pig-LV tissue protein purification, Western blotting, and imaging quantification have been described previously [15]. Membranes were incubated overnight at 4 °C with primary antibodies against cytosolic PLA_2_ (cPLA_2_), TXA_2_ synthase, and TP receptors (abcam, Cambridge, UK). After washing with TBST, membranes were incubated with the appropriate secondary antibody conjugated to horseradish peroxidase. All membranes were also incubated with GAPDH (glyceraldehyde-3-phosphate) or alpha-tubulin (Cell Signaling) for loading controls.

### Measurement of PLA_2_ activity

Left ventricular heart tissue (100 mg) was dissected and washed with PBS containing 0.16 mg/ml heparin to remove any red blood cells and clots. Tissue was homogenized in 1 ml of cold buffer (50 mM HEPES, pH 7.4, containing 1 mM EDTA). After centrifuge at 10,000×*g* for 15 min at 4 °C, the 10 μl supernatant was used for assay according to the manufacturer’s protocol.

### Chemicals

Serotonin, U46619, quinacrine, indomethacin, and SQ29548 were obtained from Sigma-Aldrich and dissolved on the day of the study.

### Data analysis

Data are presented as the mean and standard deviation (SD) of the mean. Microvessel responses are expressed as percent relaxation of the pre-constricted diameter. Microvascular reactivity was analyzed using 2-way repeated-measures ANOVA with a post hoc Bonferroni test. (GraphPad Software, Inc, San Diego, CA). A growth model was used to test the degree to which treatment groups and MetS affected the degree to which 5-HT-induced vasoconstriction, *P* values < 0.05 were considered significant.

## Results

### Pig model of MetS

Phenotypic characteristics of lean and MetS pigs are shown in Table 1. Compared to their counterparts, pigs with MetS exhibited significant increases in body weight, total cholesterol, HDL, LDL/VLDL, triglyceride, blood insulin, and blood glucose.

**Table 1:** Pig characteristics

Characteristics	Lean	MetS	*P* values
Age (weeks)	20 weeks	20 weeks	NS
Male	6	6	NS
Body weight (kg)	64 ± 15	75 ± 13	0.02
Blood insulin (pmol/L)	28.5 ± 7.1	38.6 ± 6.8	0.0002
Blood glucose (mg/dL)	92 ± 17	165 ± 35	0.0001
Total cholesterol (mg/dl)	1.12 ± 0.4	6.7 ± 1.7	0.0001
HDL (µg/µl)	0.46 ± 0.1	1.32 ± 0.3	0.001
LDL/VLDL	0.44 ± 0.1	2.72 ± 0.4	0.001
Triglycerides (mg/dl)	59 ± 34	116 ± 14.9	0.01

*HDL* high-density lipoprotein, *LDL* low-density lipoproteins, *VLDL* very low-density lipoproteins (VLDL), mean ± SD

### Increased coronary arteriolar constriction to serotonin and U46619 in the MetS pig

**Fig. 1:**
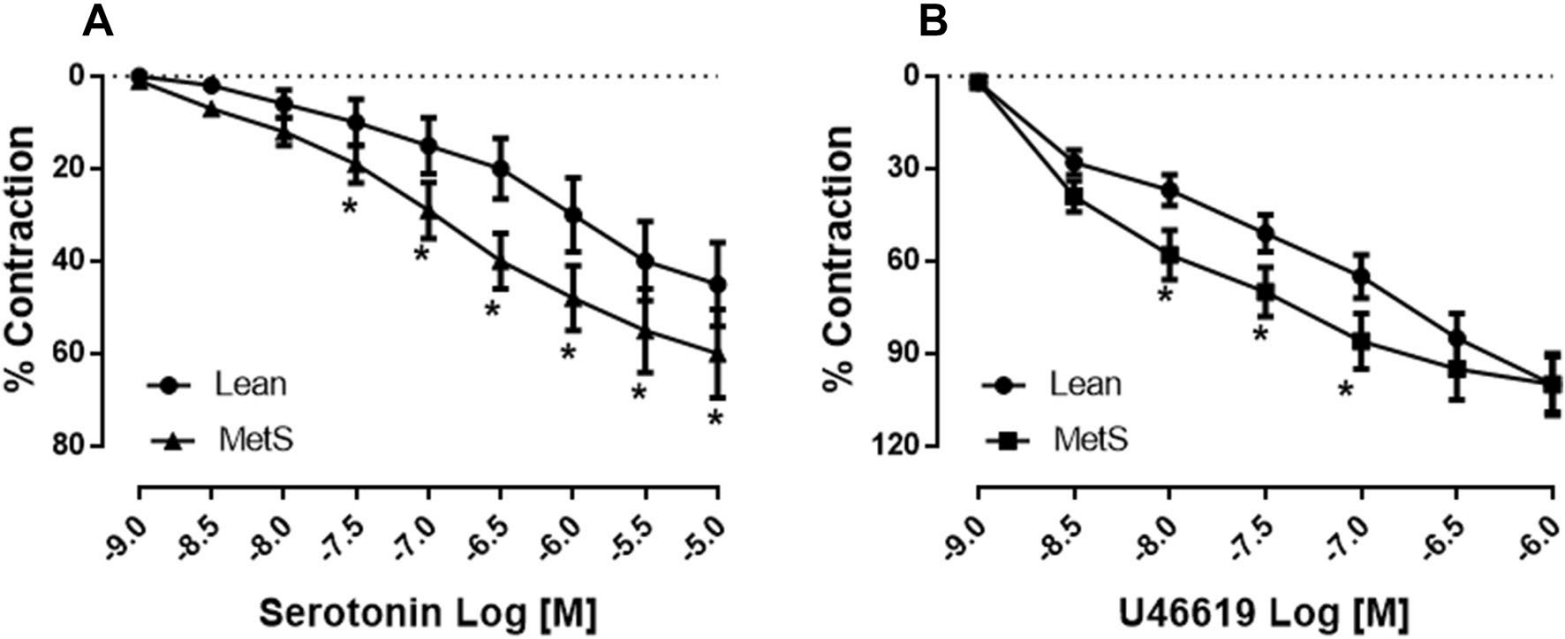
Dose-dependent contractile response of coronary-resistant arterioles to serotonin (10^−9^–10^−5^ M) and U46619 (10^−9^–10^−6^ M) in control (lean) pigs and pigs with MetS, respectively; **a** Coronary arteriolar contractile response to serotonin (10^−9^–10^−5^ M) in the non-MetS control (lean) vessels and in MetS vessels,*P < 0.05 versus lean; **b** Contractile response to U46619 (10^−9^–10^−6^ M) in the lean and MetS vessels. *P < 0.05 versus lean

Both serotonin (10^−9^–10^−5^ M) and U46619 (10^−9^–10^−6^ M) induced dose-dependent contractions of coronary-resistant arterioles in both control (lean) pigs and in pigs with MetS. These effects were more pronounced in the MetS vessels as compared with those of the control group (lean), with a 60% increase of contraction to serotonin (10^−5^ M, Fig. 1a, *P* = 0.01 vs. lean) and an 86% increase to U46619 (10^−7^ M, Fig. 1b, *P* = 0.001 vs. lean) in MetS vessels compared to the 45% contraction to serotonin and 65% to U46619 in the control (lean) vessels, respectively.

### The blockade of PLA_2_, COX, and TXA_2_ on serotonin-induced contraction

**Fig. 2:**
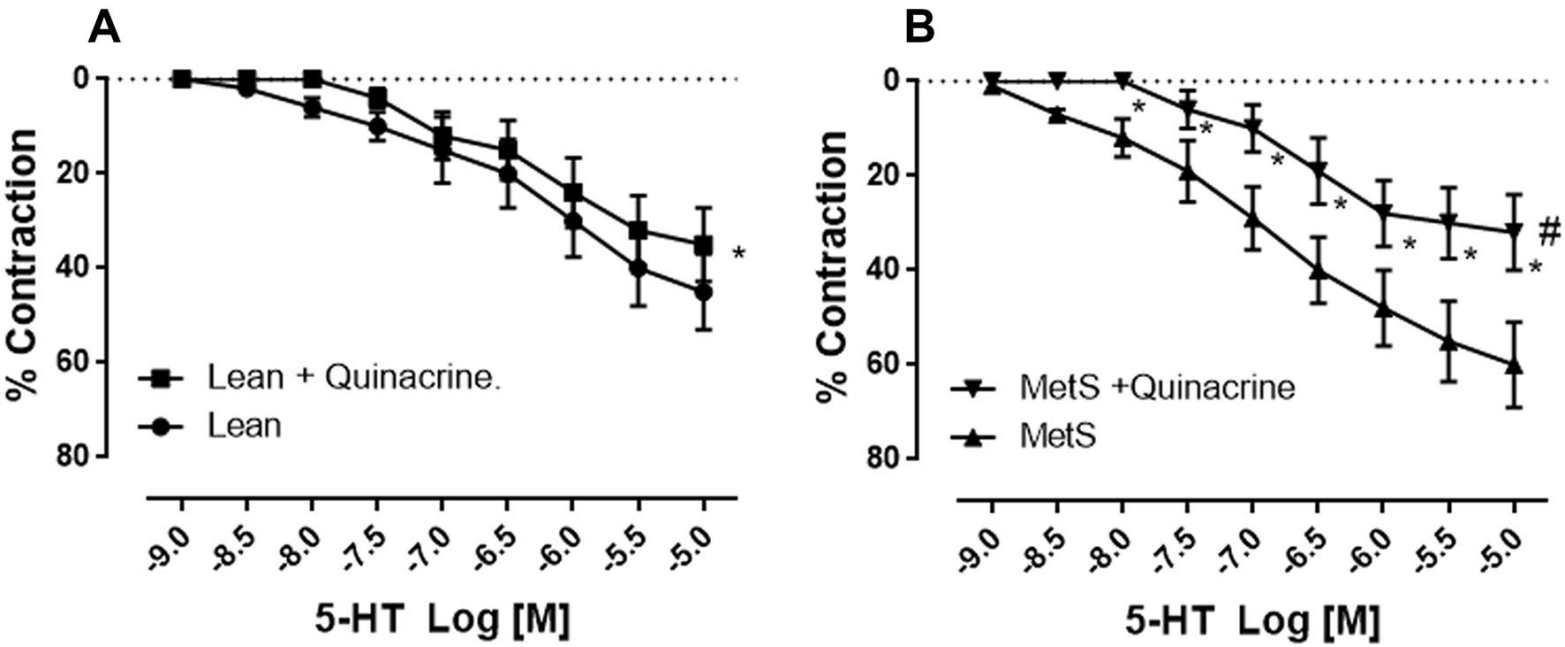
Administration of the PLA_2_ inhibitor quinacrine (10^−6^ M) significantly inhibited serotonin (5-HT)-induced contraction in both the non-MetS control (lean, **a**) and MetS vessels [*P < 0.05 vs. 5-HT alone of non-MetS control (lean, **a**) or vs. 5-HT alone of MetS (**b**)]; ^#^*P* < 0.05 versus non-MetS control (lean)

**Fig. 3:**
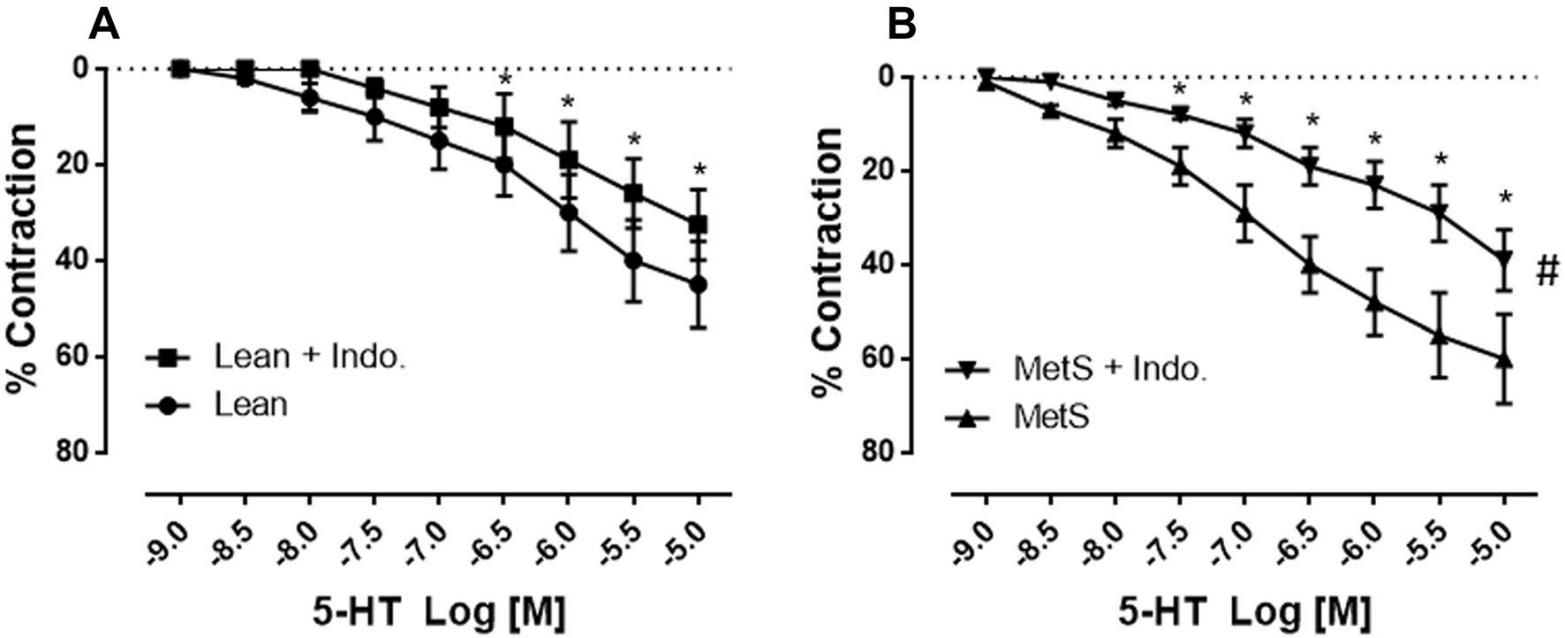
Administration of the COX inhibitor indomethacin (Indo) significantly reduced serotonin (5-HT)-induced contraction in both the non-MetS control (lean, **a**) and MetS vessels (**b**) (*P < 0.05 vs. 5-HT alone of non-MetS control (lean) or vs. 5-HT alone of MetS). ^#^*P* < 0.05 versus non-MetS control (lean)

**Fig. 4:**
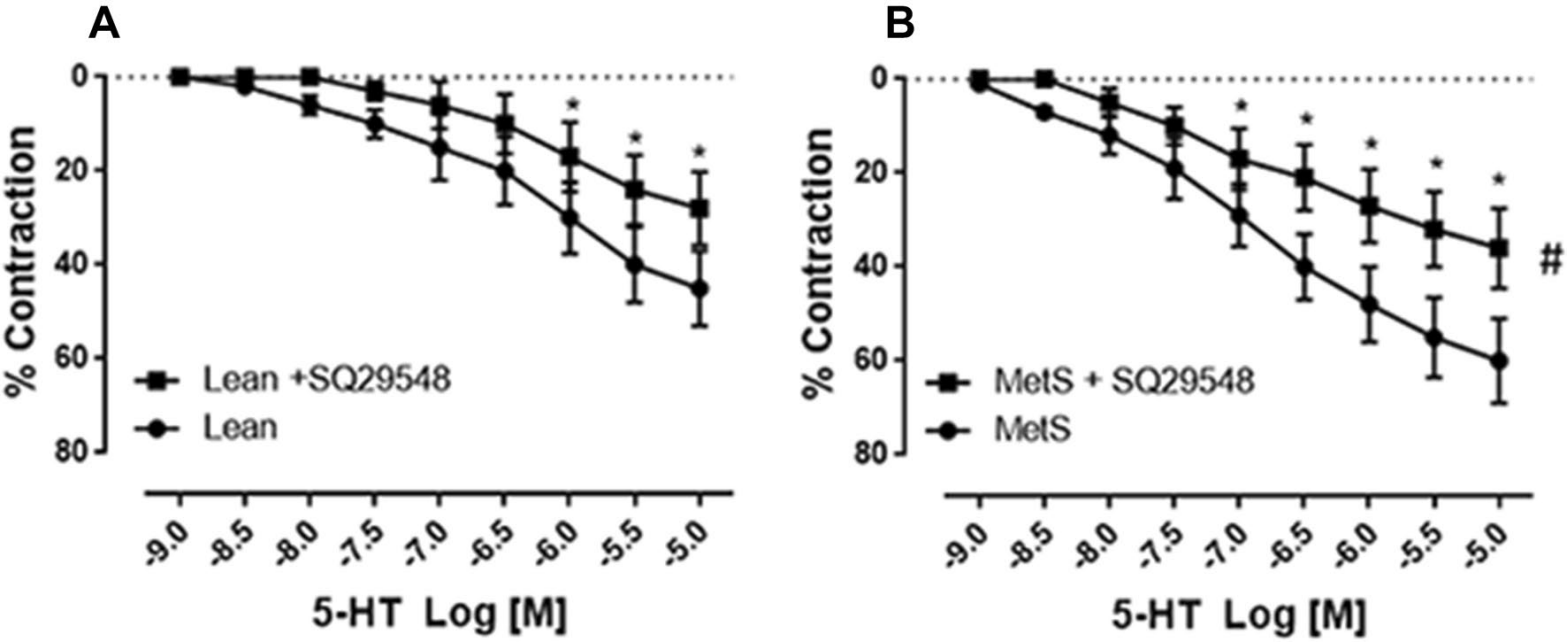
Pretreatment of the vessels with the selective thromboxane-prostanoid (TP) receptor antagonist SQ29548 significantly diminished serotonin (5-HT)-induced contraction (*P < 0.05 vs. 5-HT alone of non-MetS control (lean), or vs. 5-HT alone of MetS). ^#^*P* < 0.05 versus non-MetS control (lean)

Pretreatment of the MetS and control (lean) vessels with selective PLA_2_, COX, and TXA_2_ inhibitors significantly diminished serotonin-induced coronary arteriolar contraction (Figs. 2, 3, 4). Specifically, administration of the selective PLA_2_ inhibitor quinacrine (10^−6^ M) significantly inhibited serotonin-induced contraction in both the MetS and non-MetS control vessels (*P* = 0.002 vs. serotonin alone of non-MetS control, *P* = 0.004 vs. serotonin alone of MetS, Fig. 2). Inclusion of the COX inhibitor indomethacin (10^−5^ M) also significantly reduced serotonin-induced contraction in both the MetS and non-MetS control vessels (*P* = 0.001 vs. serotonin alone of non-MetS control, *P* = 0.003 vs. serotonin alone of MetS, Fig. 3). Furthermore, pretreatment of the vessels with the selective thromboxane-prostanoid (TP) receptor antagonist SQ29548 also significantly inhibited serotonin-induced contraction (*P* = 0.001 vs. serotonin alone of non-MetS control, *P* = 0.002 vs. serotonin alone of MetS, Fig. 4). Finally, there were significant differences in the vasoconstrictive responses to serotonin compared to the relative degree of change induced by the selective COX, PLA_2_, and TP receptor inhibitors between control (lean) and MetS groups, respectively (*P* < 0.05 vs. Lean alone, Figs. 2, 3, 4).

### Protein expression of PLA_2_, TXA_2_ synthase, and TP receptors in the pig myocardium

**Fig. 5:**
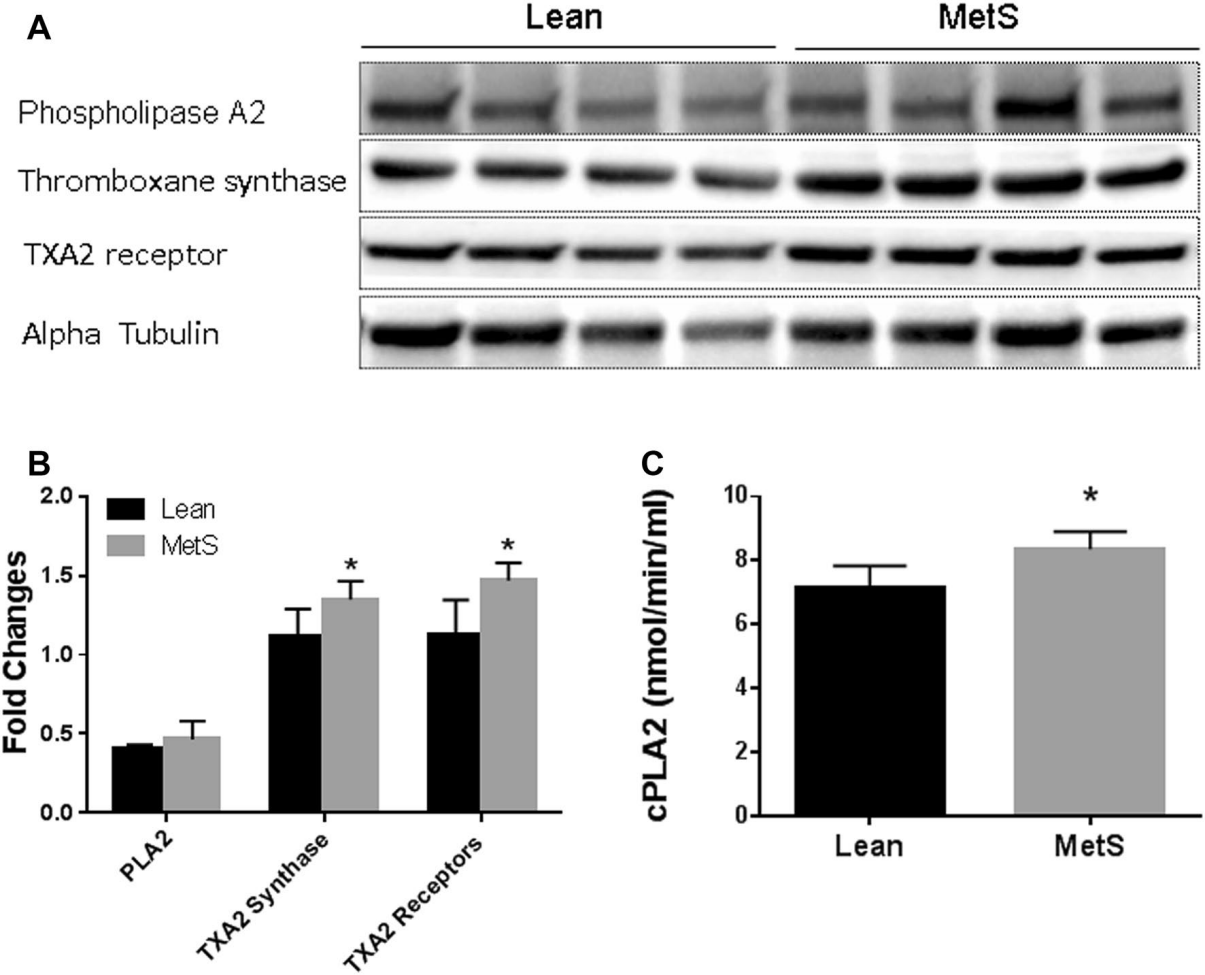
**a** Representative immunoblots of pig left ventricular tissues. Lanes 1–8 loaded with 40 µg protein were developed for PLA_2_, TXA_2_ synthase, and TP receptors; **b** Immunoblot band intensity and analysis; **c** Bar graph showing cytosolic PLA_2_ (cPLA-2) activity of the pig myocardium in the 2 groups; *n* = 4–6/group; mean ± SD. *P < 0.05 versus lean

There were no significant differences in the tissue levels of PLA_2_, between the control lean and MetS groups (Fig. 5). However, MetS exhibited significant increases in tissue levels of TXA_2_ synthase and TP receptors (*P* < 0.05 vs. lean, Fig. 5), respectively.

### PLA_2_ activity

MetS significantly increased the PLA_2_ activity in the LV myocardium compared with normal diet control group (lean) (*P* < 0.05, Fig. 5c).

## Discussion

There are a number of novel findings in the current study. First, early stage of MetS caused an increased contractile response of coronary arterioles to serotonin in the juvenile pig. Second, the enhanced responses to serotonin were significantly prevented in the presence of PLA_2_, COX, and TXA_2_ inhibitors. Finally, MetS induced activation of PLA_2_, and protein overexpression of TXA_2_ synthase/TXA_2_ receptors of heart tissue in the juvenile pigs.

MetS impairs endothelium-dependent relaxations to several platelet-derived substances in coronary microvasculature [10, 12]. MetS also markedly alters the response of coronary microcirculation to serotonin and TXA_2_ in a monkey model [12]. In most blood vessels, if the smooth muscle cells are exposed to serotonin, vasoconstriction occurs following activation of 5-HT receptors [15, 16]. We have recently found that the coronary arteriolar contractile response to serotonin was further altered after chronic myocardial ischemia in pigs with MetS [21, 22, 24, 25].

In the current study, we further observed that MetS without myocardial ischemia significantly increased constriction of coronary arterioles to serotonin and the TXA_2_ analog U46619 in the juvenile pig. This finding indicates that early-stage MetS may cause coronary arteriolar spasm, resulting in downregulation of myocardial perfusion.

The mechanisms responsible for serotonin-induced coronary constriction have been studied by our group and other investigators [12, 15, 16, 21–23, 27]. We have previously found that myocardial ischemia/reperfusion causes PLA_2_ expression/activation, which contributes to serotonin-induced coronary vasoconstriction in patients after CP/CPB and cardiac surgery [15, 16]. In the present study, we found that serotonin-induced coronary arteriolar constriction was prevented by the selective PLA_2_ inhibitor quinacrine, suggesting that activation of PLA_2_ contributes to serotonin-induced vasoconstriction in the setting of early MetS.

High-fat diet alters lipid profiles, which may lead to a pro-inflammatory state of the vascular wall and increases the risk of coronary heart disease. Changes in the arachidonic acid (AA) metabolism via COX may affect coronary function in MetS and obesity in a rodent model [23]. We have found that COX inhibition in pigs with chronic myocardial ischemia and hypercholesterolemia improves coronary microvascular function without effects on collateral-dependent territory [21, 22]. We and others have also demonstrated that diabetes and cardioplegic ischemia upregulate COX expression in the human myocardium and coronary microvasculature, which contributes to regulation of coronary microcirculation [15, 16, 22, 24, 25]. The current study further demonstrates that serotonin-induced vasoconstriction was enhanced in the MetS microvessels and inhibited in the presence of the COX inhibitor indomethacin in the non-ischemic heart tissue/vessels, suggesting that MetS regulation of serotonin induced constriction via activation of COX in the juvenile pigs.

Arachidonic acid is mainly metabolized to vasoconstrictor prostanoids, including TXA_2_ via COX in coronary arterioles from animals and humans [15, 16, 22–25]. We and others have previously reported that diabetic and ischemic upregulation of TXA_2_ contributes to modification of coronary arteriolar vasodilation and vasoconstriction in humans [15, 16, 22–25]. A high-fat diet impairs tissue perfusion in ischemic myocardium of naproxen-treated swine by shifting the prostanoid balance to favor production of TXA_2_ over PGI_2_ [22, 24, 25]. Furthermore, in the present study, we observed that the selective TP receptor inhibitor markedly diminished the coronary arteriolar contraction to serotonin in the pig model of MetS alone in the absence of chronic myocardial ischemia and drug pretreatment. In support of our physiological study, we also observed that early-stage MetS is associated with increased PLA_2_ activity protein expression of TXA_2_ synthase and TP receptors in the pig heart tissues.

Impaired microvascular insulin signaling may develop before overt indices of microvascular endothelial dysfunction and represent an early pathological feature of adolescent obesity [26]. Microvascular insulin resistance and endothelial dysfunction in skeletal muscle, brain, and heart occur early in the development of juvenile obesity in pigs [26, 27]. In the current study, we further observed the enhanced vasomotor tone of the coronary microcirculation in 5-month-old juvenile pigs with MetS. These novel findings suggest that coronary microvascular dysfunction is therefore an early manifestation of obesity/MetS and might contribute to the increased cardiovascular risk and can partially explain the reduced coronary flow reserve and increased minimal vascular resistance in patients with MetS. Thus, the present study paves the way for finding new strategies to improve prevention of complications of early pediatric obesity [28, 29].

In conclusion, these results indicate that MetS is associated with the increased contractile response of porcine coronary arterioles to serotonin, which is in part via upregulation/activation of PLA_2_, COX and subsequent TXA_2_. These novel findings suggest that the alteration of coronary arteriolar vasomotor function may occur during early stages of metabolic syndrome and juvenile obesity.
